# Computational pharmacokinetics at a crossroads

**DOI:** 10.1186/2193-9616-1-5

**Published:** 2013-03-06

**Authors:** Frédéric Y Bois

**Affiliations:** DRC/VIVA/METO, INERIS and Chair of Mathematical Modeling for Systems Toxicology, Technological University of Compiegne, Compiegne, France

**Keywords:** Pharmacokinetics, Modeling, Editorial

## Abstract

This first special issue of *In Silico Pharmacology* focuses on computational pharmacokinetics since they are an important part of integrated applications in computational pharmacology. The important topics of model structure, model parameterization, improved organ description, and modeling of drug-drug interactions are covered. They are actually at the crossroads between several emerging disciplines which will shape the future of therapeutic treatments and public health.

## Purpose

We have chosen to focus this first special issue of *In Silico Pharmacology* on computational pharmacokinetics. In our view, that was a logical step since pharmacokinetics are a foundational part computational pharmacology.

## Main text

Pharmacokinetic (PK) modeling has come quite a long way since it started in the fifties, to the point that we know call it "computational pharmacokinetics"… From a branch of (statistical) modeling applied to PK, it has become a sub-discipline of PK itself. The drive for that mutation has clearly been the need to exceed the limits of interpolation and parameter estimation provided by statistical models (even if they were biologically motivated, as compartment models can be) and to enter the realm of extrapolations and predictions. That was a main point of the last Paris talk given by the much regretted L. Sheiner, a few years ago already.

Physiologically-based PK modeling (, (PBPK) is at the heart of this evolution. Originally developed as a conceptual tool Teorell, *e.g.*^1937^, (), applied for interspecies extrapolation Dedrick *et al.*^1973^), incubated and refined for quite a while in the data poor environment of environmental toxicology, it has really taken off during the last 10 years when specialized software and parameter databases were made available. This issue of In Silico Pharmacology offers clear illustrations of some of the most active developments in this area.

At the heart of PBPK modeling, we find developments in model structure and parameterization. Mastering the various concepts and techniques used in PBPK modeling is a challenge. The whole gamut of differential equation development and solving, and practically all of the numerical computation techniques, up to advanced stochastic simulatios, are relevant. The major advantages, for practitioners, of pre-coded general PBPK models such as PKSim® (developed by Bayer Technology®) or Simcyp® are obvious: standardization, yet flexibility, ease of use, hiding from user the ( at Simcyp® Jamei, (usually) unnecessary computational details, focusing on substantive questions rather than spending time of coding and solving. Yet, biology, or even PK, cannot be subsumed to the existing models and an unlimited number of improvements are possible. How to manage those, in a quality assurance framework, is the major topic of the article by M. Jamei *et al.*^2013^, (.While waiting for the perfect model and databases, the developers of PKSim® illustrate the power of Bayesian population analyses in the context of PBPK modeling. Getting improved parameter distributions, helping clinical data analysis, and even making substantive inference and actual discovery about a biological process are all demonstrated Krauss *et al.*^2013^, ( It is to me a great pleasure to see our early contribution Gelman *et al.*^1996^) to this line of research finally comes of age.

At the frontiers of PBPK modeling lie two vast research areas: virtual organ models and systems biology. Only the future will tell us whether those two fields merge and what happens to "PBPK" modeling *per se* in that case… Meanwhile, we can probably usefully borrow from the developments in both those areas. It would actually be somewhat illogical to invest in pharmacokinetic research at the cell level without paying attention to the way cells are organized in tissues and organs. To pharmacokinetics, as sensitivity analyses tend to show, the most important organ models to develop are those related to inputs and outputs (,the A, M and E in ADME). This journal is expected to cover extensively all those in the near future. Virtual organ models can also receive specific applications in local pharmacokinetics, physiology or pharmacology, without linking to PBPK. This special issue features an important article on the coupling of a detailed skin model to a PBPK model by Procter and Gamble® research laboratories Dancik *et al.*(2013,). It also illustrates one of the most important and sought for application of PBPK modeling Adler *et al.*(2011): Quantitative *in vitro* to *in vivo* extrapolation (QIVIVE). This is also a topic of the review paper by N. Quignot Quignot, (2013, in the context of blood to tissue barriers, and of the last paper from A. Owen's group Siccardi *et al.*(2013). Quignot's paper actually returns us back at some foundational issues in PBPK modeling. In a way, barriers (and inter-molecular interactions) are such stuff as PBPK models are made on. The question of barriers, their identification and models, is in a sense a prerequisite to PBPK modeling. This question is actually pressing for the proper construction of such models for nanocarriers, for example.

Siccardi *et al.* paper, besides QIVIVE, presents another important application of PBPK modeling: predicting drug-drug interactions. This is directly linked to out last frontier: systems biology. Metabolism has always been the Achilles' heel of PBPK models. I have rarely seen serious criticisms of a PBPK model but in that grey area where sophisticated transport mechanistic modeling meets the most simplistic assumptions about metabolism (linear *vs.* Michaelis-Menten for short). The situation has markedly improved during the last 10 years, for example in Simcyp®, but the interface between a drug (and *a fortiori* several concurrently) and the molecular cell machinery, be it for active transport, metabolism and early effects is a totally open question and any advance in the area is most welcome. The PKSim® sofware has a flexible answer (MoBi®), and an alternative route via systems biology markup language (SBML) models composition has been proposed by us Bois, (2009). These developments are very exciting as PBPK models are much called for in systems biology models for handling circulating species.

## Conclusion

Why "at a crossroads" in the title of this commentary? PBPK modeling and computational pharmacokinetics in general are, I think, approaching a "tipping point" at which they can merge with either or both virtual body modeling or systems biology. We may well be at the very place where the merging of all three will happen, for a better understanding and predictions of our underlying biology. I remember gazing with much curiosity and excitment the covers and titles of "Theoretical Biology" during my freshman year's. This was beyond my reach at the time… But the excitement is still going on after all these years, with added fun and a sense of progress. I am convinced that the contributions to this journal will continue to convey this ambivalent sense of both discovery and achievement.
